# The role of E255K/V-inclusive mutations in a Philadelphia-positive acute lymphoblastic leukemia with mutation evolution during sequential TKIs therapies: A case report

**DOI:** 10.1097/MD.0000000000025579

**Published:** 2021-05-07

**Authors:** Miaomiao Zhao, Xiya Gui, Qiuling Wu, Linghui Xia, Yadan Wang

**Affiliations:** a Institute of Hematology, Union Hospital, Tongji Medical College, Huazhong University of Science and Technology, Wuhan; b Department of Hematology, People's Hospital of Hanchuan, Wuhan University, Hanchuan, Hubei, China.

**Keywords:** BCR-ABL1 mutation, clonal evolution, compound mutation, E255K/V-inclusive mutation, Philadelphia chromosome-positive acute lymphoblastic leukemia, ponatinib

## Abstract

**Rationale:**

Until recently, the survival rate in patients with Philadelphia-positive acute lymphoblastic leukemia (Ph+ ALL) was approximately 30%. Tyrosine kinase inhibitors (TKIs), which are a new class of drugs that target BCR-ABL fusion protein, have shown to be effective in treating Ph+ ALL in adults. However, the resistance mechanisms that promote the disease recurrence have altered the initial success of these revolutionary agents.

**Patient concerns:**

A 71-year-old Chinese female patient who suffered from severe shoulder and back pain for 1 week.

**Diagnosis:**

The patient was diagnosed with Ph+ ALL (B–cell) because of the following items. Complete blood count showed extremely abnormal white blood cell count (26.26×10^9^/l), hemoglobin concentration (65 g/l) and platelet count (14×10^9^/l). And because that Bone marrow aspirate showed 72.5% lymphoblasts and 59.30% lymphoblasts were confirmed by flow cytometry (FCM). At mean time, Real-time fluorescent quantitative PCR analysis confirmed that the P190 BCR/ABL fusion gene expression was 5.9%. Karyotype analysis indicated the following: 45, XX, −7, t (922) (q34; q11) [cp3].

**Interventions:**

The patient was treated with chemotherapy and different TKIs including imatinib, dasatinib, ponatinib, and bosutinib.

**Outcomes:**

The patient achieved complete remissions with different TKIs after diagnose but relapsed afterward and died of infection.

**Lessons:**

Multidrug-resistant mutations within the BCR-ABL1 kinase domain are an emerging clinical problem for patients receiving sequential TKIs therapy. Acquisition of E255K/V-inclusive mutations is usually associated with ponatinib resistance, thus it is necessary to screen out new real pan-inhibitor compounds for all BCR/ABL mutations and figure out the potential efficacy of asciminib-based drug combinations in the future.

## 1 Introduction

Philadelphia-positive acute lymphoblastic leukemia (Ph+ ALL) is a rare subtype of ALL characterized by enlarged spleen, liver and lymph nodes, paleness, fevers, bruising, bone pain, abnormal blood cell counts, and weight loss. Until recently, the survival rate in patients with Ph+ ALL was approximately 30%.^[1]^ Yet, with the recent discovery of a new type of drugs, that is second- and third-generation Tyrosine kinase inhibitors (TKIs), which specifically target the Philadelphia chromosome, the survival rates have doubled. Recent clinical trials suggest that TKIs are more effective for elderly patients who cannot obtain a good outcome from intensive chemotherapy or allogeneic stem cell transplantation.^[1]^ However, genetic instability fostering the acquisition of BCR-ABL1 kinase domain mutations is high, thus increasing the likelihood of subsequent TKI-resistant relapses.^[2]^

Prompt therapeutic reassessment and individualization based on mutation status are important to regain response and prevent disease progression.^[1]^ It is important to differentiate compound mutations from polyclonal mutations in order to come up with the best treatment option. A risky element of sequential TKI treatment is the selection of BCR-ABL1 compound mutants, defined as harboring ≥2 mutations in the same BCR-ABL1 allele that have the potential to confer resistance to multiple TKIs.^[3]^

Here we reported a single case of an elderly female patient with Ph+ ALL who achieved complete remissions with different TKIs after diagnose but relapsed afterward and died of recurrence, that is resistance to ponatinib and bosutinib caused by the burst of multiple mutations including E255K/V. We revealed the mutation evolution during sequential TKI therapies by monitoring BCR-ABL kinase domain mutations using next-generation sequencing (NGS). This study provided strong evidence that the mutation evolution of the BCR-ABL kinase domain can be a key factor influencing the treatment decision making in elderly Ph+ ALL patients. We also discovered that the E255K/V-inclusive mutations may be a dangerous indicator of resistance to ponatinib.

## 2 Case report

**Figure 1:**
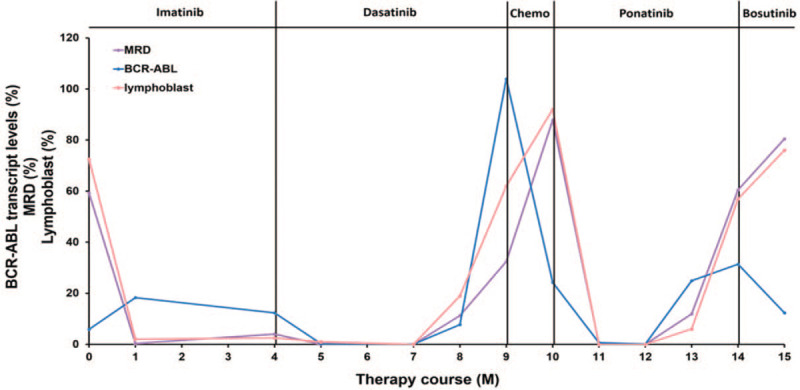
Dynamic changes of MRD, BCR-ABL, and lymphoblast during TKI therapy. The purple line represents minimal residual disease (MRD) detected by flow cytometry; the blue line represents the level of BCR-ABL transcript; the pink line represents the percent of lymphoblast in the bone marrow.

A 71-year-old Chinese female patient who suffered from severe shoulder and back pain for 1 week was admitted to our hospital on January 30, 2017. Complete blood count showed extremely abnormal white blood cell count (26.26 × 10^9^/l), hemoglobin concentration (65 g/l) and platelet count (14 × 10^9^/l). Bone marrow aspirate showed 72.5% lymphoblasts (Fig. 1). Consequently, 59.30% lymphoblasts were confirmed by flow cytometry (FCM) (Fig. 1). Real-time fluorescent quantitative PCR analysis confirmed that the P190BCR/ABL fusion gene expression was 5.9% (Fig. 1). Karyotype analysis indicated the following: 45, XX, −7, t (9,22) (q34; q11) [cp3]. The patient had no history of other diseases, except moderate splenomegaly and hypertension. Moreover, the examination of cerebrospinal fluid (CSF) taken by lumbar puncture confirmed no involvement of the central nervous system. As a result, the patient was diagnosed with Ph+ ALL (B–cell).

**Figure 2:**
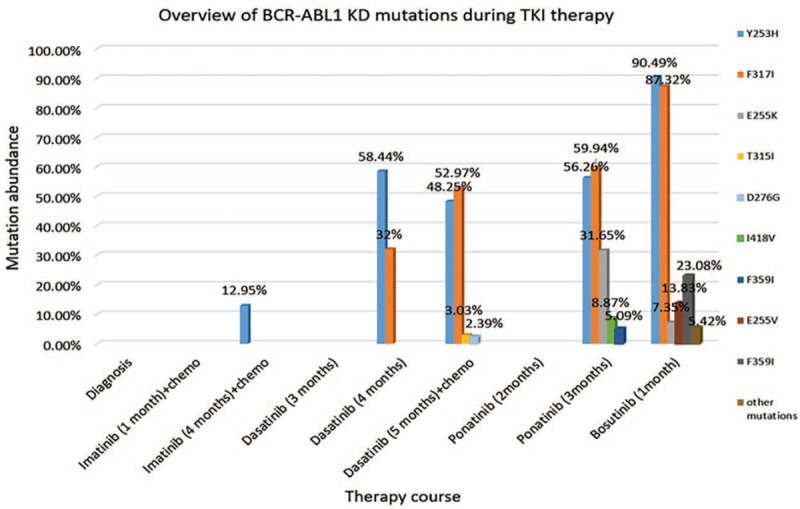
Overview of the clonal evolution of BCR-ABL kinase domain mutations during TKI therapy. Mutated population abundances in relation to therapeutic intervention during different time points. At diagnosis, there was no point mutation; the point mutation disappeared after 1-month therapy with Imatinib and chemotherapy, after 3-months of therapy with Dasatinib, and after 2-months therapy with Ponatinib. The point mutations increased after 4-months of therapy with Imatinib + chemotherapy, after 4-months therapy with Dasatinib, after 5-months therapy with Dasatinib + chemotherapy, after 3-months of therapy with Ponatinib, and 1-month therapy with Bosutinib. Other mutations included I418 V (3.39%) and D276G (2.03%).

On February 9, 2017, the patient was treated with conventional chemotherapy, that is DVP, which included daunorubicin (40 mg/d) for the first 3 days, vindesine (4 mg/d) on days 1, 8, 15, and 22, and prednisolone (1 mg/kg/d for the first 14 days and 0.5 mg/kg/d for the next 14 days), along with oral imatinib (400 mg/d) treatment. At the same time, the patient received platelet transfusion, rehydration, and anti-infective treatment. Soon after the DVP treatment course, the patient's abnormal clinical symptoms disappeared. Blood routine examination, bone marrow smear, and FCM testing showed that all indicators were within the normal range. Even though the expression of the *BCR-ABL* gene was still positive, with 18.3% (Fig. 1), this patient had obtained a complete hematological response. This patient was prescribed oral imatinib (400 mg/d) for another 3 weeks. During the following month, the patient was treated with vindesine and prednisolone (vindesine 4 mg/d on days 1, 8, 15, and 22, and prednisolone 1 mg/kg/d for the first 14 days and 0.5 mg/kg/d for the next 14 days) and oral imatinib (400 mg/d) because of the severe pulmonary infection that appeared during the initial chemotherapy. After 4 months of imatinib therapy, bone marrow aspirate showed 2.5% lymphoblast the minimal residual disease (MRD) detection by FCM was 4.04%, and the level of the *BCR-ABL* gene expression decreased to 12.32% (Fig. 1). However, NGS showed Y253H (12.95%) point mutation (Fig. 2) in the BCR-ABL kinase domain, which indicated imatinib resistance.^[4]^

According to the protocol schedule, the patient was treated with DVP chemotherapy and oral dasatinib (100 mg/d) from June 5, 2017. One month later, bone marrow aspiration showed 1% lymphoblast, MRD was significantly reduced to 0.0026%, the level of *BCR-ABL* gene expression dropped to 0.28%, and no mutation was found in the BCR-ABL kinase domain (Fig. 1). The patient continued to take dasatinib orally without any other therapy for 3 months. No mutation in the BCR-ABL kinase domain was found in the first 2 months. However, during the last month of single oral dasatinib treatment (October 20, 2017), the patient experienced shoulder and back pain along with headaches. Bone marrow smear indicated 19% lymphoblast, MRD was 11.28%, and the expression of the *BCR-ABL* gene was 7.75% (Fig. 1). New point mutation F317I (32%) was found in the BCR-ABL kinase domain along with Y253H (58.44%) by NGS (Fig. 2). Meanwhile, lots of lymphoblasts found by CSF smear and FCM of CSF confirmed lymphoblast involvement of the central nervous system. The patient was then treated with dasatinib (100 mg/d) and chemotherapy consisting of methotrexate (1 g day 1), idarubicin (5 mg day 2), dexamethasone (5 mg day 1–day 5), temozolomide (200 mg day 3–day 7), along with lumbar puncture and intrathecal cytarabine (50 mg) and methotrexate (10 mg) injection twice a week. Although her CSF was clear without any lymphoblast, 2 weeks after chemotherapy, bone marrow smear indicated 62% lymphoblast and MRD was 32.43% (Fig. 1). The level of BCR-ABL transcript increased to 103.96%, and multiple point mutations were found in the BCR-ABL kinase domain including Y253H (48.25%), F317I (52.97%), T315I (3.03%), D276G (2.39%), and V289A (0.51%) (Fig. 2).

On December 17, 2017, the patient was treated with oral ponatinib (45 mg/d), which is the recommended treatment option for patients with the T315I mutation. During the first 2 months’ therapy of ponatinib, her blood routine examination had a significant improvement, and her pain symptoms were obviously alleviated at the beginning of therapy. The patient had another complete remission since lymphoblast, MRD, and the expression of the *BCR-ABL* gene decreased to normal level. Nevertheless, in March 2018, the patient relapsed, 6% lymphoblast were found in the bone marrow, MRD increased to 11.89%, the expression of *BCR-ABL* gene increased to 24.95% (Fig. 1). And various point mutations appeared in the BCR-ABL kinase domain including Y253H (56.26%), F317I (59.94%), E255K (31.65%), F359I (5.09%), and I418 V (8.87%) (Fig. 2). After additional treatment with ponatinib (45 mg/d) for 20 days, bone marrow aspiration indicated increased lymphoblast. Furthermore, the karyotype analysis was as follows: 45, XX, −7, t (9, 22) (q34; q11) [9]/46, XX [11]. The patient was not eligible for enrollment in the clinical trial of cellular immunotherapy because of pulmonary infection. Moreover, blinatumomab, an anti-CD19/CD3 bispecific antibody, is currently not available in China.

Consequently, the patient was treated with Bosutinib only (500 mg/d for 1 week) or in combination with standard chemotherapy (vincristine 2 mg per day DAY 1, 8; prednison 60 mg per day DAY1–DAY14); however, no improvements were observed. One month later (May 8, 2018) her bone marrow smear showed 76% lymphoblast, the MRD was up to 80.47%, the expression of *BCR-ABL* gene was 12.31% (Fig. 1) and more various point mutations appeared in the BCR-ABL kinase domain, including Y253H (90.49%), F317I (87.32%), E255K (7.35%), E255 V (13.83%), F359I (23.08%), I418 V (3.39%), and D276G (2.03%) (Fig. 2). Unfortunately, the patient suffered severe pulmonary infection again and died on progressing disease 1 month after. Her overall survival time was 1 year and 4 months.

In order to clarify the role of compound mutations for ponatinib and bosutinib resistance, we analyzed all the NGS data regarding the ABL domain mutation retrospectively. The NGS technology offers high sensitivity, but it cannot be used to discriminate between polyclonal and compound mutations. The average read length is between 200 to 300 bp, which could not cover all the mutation sites on the ABL kinase domain, except site E255 and site Y253. The data of the last 2 detections after ponatinib resistance showed that all E255K or E255 V mutations occurred on the Y253H mutation sequence reads. Finally, The Y253H/E255K (V) compound mutations were confirmed and the presence of other compound mutations was inferred by the detection of 2 mutations with a combined frequency >100% (e.g., 56.26% Y253H and 59.94% F317I) according to reference^[5]^ (Table 1).

**Table 1:** The frequency of compound mutations in BCR-ABL kinase domain.

Therapy course (month)	Therapy	Compound mutation	Frequency
13	ponatinib	**Y253H/E255K**^∗^	**31.65%**
		Y253H/F317I^†^	U
15	bosutinib	**Y253H/E255V**^∗^	**13.83%**
		**Y253H/E255K**^∗^	**7.35%**
		Y253H/F317I^†^	U
		Y253H/F359 I^†^	U
		F317I/F359I^†^	U

U = undetermined. Bold is in order to clarify the role of compound mutations for ponatinib and bosutinib resistance.

∗ Compound mutations, which were confirmed by NGS due to they are in the same amplified reads.

† Compound mutations, which were inferred by the detection of 2 mutations with a combined frequency >100%, according to reference^[5]^.

**Figure 3:**
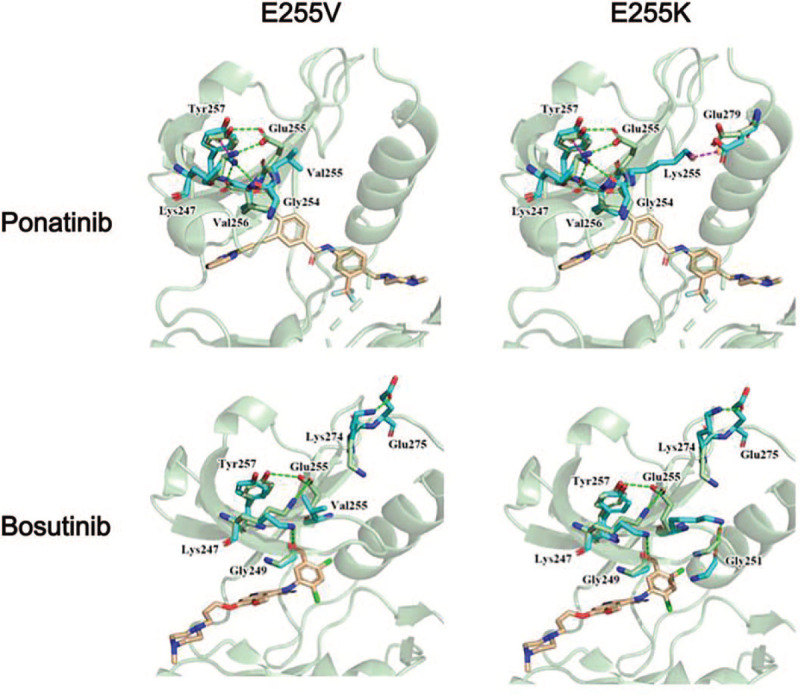
The structural changes caused by the resistance of single point mutations (E255 V and E255K) to ponatinib and bosutinib. TKIs were displayed in wheat sticks. ABL kinase is illustrated in pale green cartoon representation, and the interacting residues are labeled and shown with green sticks (wild type) and cyan sticks (mutant type). Interactions between residues and drugs are shown in dashed lines: hydrogen bondings in blue, salt bridge in red, π-π stacking in yellow, and hydrophobic interaction in gray color. **Ponatinib**: bound to the ABL1 kinase domain in the DFG-out mode, recognizing an inactive conformation of the kinase. The binding site of ponatinib is centered on the adenine pocket of the enzyme and extends from the phosphate-binding loop (P-loop) to the C-helix region. In the native protein, hydrogen bonds of Glu255 to Tyr257, and Lys247 were detected, contributing to a specific conformation of the P-loop. In E255 V mutation, the hydrogen bond with Val255 was lost. However, the Lys247 formed the hydrogen bond with the backbone oxygen atoms of both Gly254 and Val256 and formed a π-cation interaction with the benzene ring of Tyr257, which changed the original conformation of the P-loop, resulting in a certain degree of drug resistance. In the E255K mutation, Lys247 formed the same interactions with surrounding residues Gly254, Val256, and Tyr257, but Lys255 with a long side chain and protonated N atom was able to interact with Glu279 by a salt bridge, which also changed the original conformation of P-loop, resulting in drug resistance. **Bosutinib:** in the native protein, Glu255 formed hydrogen bonds with both Tyr257 and Lys247 in the P-loop region, which play an essential role in maintaining the conformation of P-loop. The mutation of E255 V brought about a loss of these 2 interactions and the orientation of Lys247 towards Gly249 forming a hydrogen bond. Moreover, Lys274 underwent a significant conformational change, producing a hydrogen bond with Glu275. These might induce a conformational change of the P-loop and affect the binding of Bosutinib. As a result, drug resistance occurs. The E255K mutation was similar to E255 V. In addition to the loss and gain of the hydrogen bonds mentioned above, Lys255 can form hydrogen bonds with Gly251 located in the turning of the P-loop. Such interaction changes might cause large conformational alteration in P-loop, leading to drug resistance.

Next, by analyzing the crystal structure and discussing some literature results, we provided the structural explanation for the resistance of single point mutations (Y253H, E255K, and E255 V) to second- and third-generation TKIs (Fig. 3 and see Fig. S1, supplemental content, which demonstrates the structure of point mutations to 4 TKIs.). The resistance of each mutation was assigned a value (RI, resistance index). We predicted the resistance of compound mutations using the sum of these indices and analyzing the resistance mechanism of compound mutation from the perspective of structure. The results showed that E255K single mutation and Y253H/E255K compound mutation conferred high- or intermediate-level resistance to 4 clinically available second- and third-generation TKIs (see Table S1, supplemental content, which illustrates the resistance indices of single and compound mutations to 4 TKIs). Besides, the E255 V single mutation and Y253H/E255 V compound mutation, which confers sensitivity to dasatinib, intermediate-level resistance to bosutinib, and high-level resistance to ponatinib, were most resistant to nilotinib.

Written informed consent was obtained from the patient's family for the publication of this case report.

## 3 Discussion

Here we report a single case of an elderly female patient with Ph+ ALL who was first treated with imatinib to dasatinib. The first complete hematological response was followed by rapid hematologic relapse, which was associated with Y253H (12.95%) and F317I (32%) point mutation, and some minor mutation T315I (3.03%), D276G (2.39%), and V289A (0.51%) point mutation. Next, the patient received ponatinib therapy; ponatinib is the only approved BCR-ABL tyrosine kinase inhibitor that is active against T315I mutation.^[6]^ After the therapy, the patient was in complete remission, and no point mutation was found in the BCR-ABL kinase domain. Nevertheless, transient response to ponatinib was followed by the rapid third relapse with a novel major mutation E255K and a compound mutation Y253H/E255K. According to the recommendation of NCCN Guidelines of Acute Lymphoblastic Leukemia (Version 2, 2019), bosutinib is considered a good option for relapsed or refractory Ph+ ALL with E255 V/K, F317L/V/I/C, F359 V/C/I, T351A, or Y253H mutations,^[4]^ which matched the ABL mutation profile at the third relapse in our patient well. Finally, the patient was treated with bosutinib but unfortunately died due to severe pulmonary infection and progressing disease.

### 3.1 The major possible reasons for ponatinib and bosutinib resistance in this patient

#### 3.1.1 Ponatinib could not overcome E255 V/K mutation-based resistance

A previous preclinical study indicated that the ponatinib IC50 values for E255K/V (IC50 14/36) were higher than that of T315I (IC50 11).^[6]^ The E255 V mutation, which confers high-level resistance to imatinib and intermediate-level resistance to nilotinib and dasatinib, was most resistant to ponatinib.^[7]^ Our computer structure analyses suggested that E255K and E255 V shift the P-loop (Fig. 3), intruding on the ponatinib binding site, which results in drug-resistance. Furthermore, Cortes et al showed that Ph+ ALL patients with E255 V/K at baseline achieved a worse response to ponatinib than chronic-phase chronic myeloid leukemia patients with the same mutation.^[5]^ All the above data suggested that ponatinib, which is an active pan-inhibitor of BCR-ABL, including the T315I mutation, might not be effective for Ph+ ALL patients with E255 V/K mutation-based resistance.

#### 3.1.2 Bosutinib may not be an optimal choice for relapsed or refractory Ph+ ALL with E255K/V mutations

The previous studies have suggested that bosutinib monotherapy may not be effective for Ph+ ALL patients, especially those carrying more than 1 mutation (E255 V, F317L, F359C).^[8,9]^ This might be the reason why the FDA has not approved Bosutinib as the first-line treatment of the Ph+ ALL.^[9]^ Moreover, Sara et al found that E255K/V mutation might be the reason for bosutinib-resistance because of high IC50 values.^[7]^ Our computer structure analyses show that E255K and E255 V induce a conformational change of the P-loop and affect the binding of Bosutinib (Fig. 3). Accordingly, for patients with relapsed or refractory Ph+ ALL, especially those with E255K/V mutations, bosutinib may not be an optimal choice.

#### 3.1.3 BCR-ABL1 compound mutants have increased the complexity of the mechanisms of resistance to TKIs

Several studies have suggested that the compound mutations are associated with the TKIs-resistance.^[10]^ For example, Zabriskie et al found that T315I-inclusive compound mutations significantly impair ponatinib binding and may lead to clinical resistance and relapse.^[10]^ In our patients, series BCR-ABL kinase domain mutations, that is Y253H/E255K and Y253H/E255 V, that were confirmed by NGS, appeared during the end-stage. Our computational modeling results indicated that Y253H/E255K confers high- or intermediate-level resistance to all clinically available TKIs. Clinical data also further confirmed the resistance of the ponatinib to this compound mutation in BP-CML and Ph+ ALL patients.^[5]^ Compared to Y253H/E255K, in vitro experimental results and computational modeling have identified dasatinib as the only clinically available TKIs that retain potency against Y253H/E255 V at clinically relevant levels.^[10]^ The superiority of dasatinib compared to ponatinib against the Y253H/E255 V compound mutant was further confirmed by our computational modeling results, which revealed that the resistance index of ponatinib is higher than that of dasatinib (2 vs 0). Other possible compound mutations (Y253H/F317I, Y253H/F359I, and F317I/F359I) could also increase the complexity of the resistance mechanisms to TKIs. It could be speculated that all these compound mutants are among the main mechanisms underlying ponatinib and bosutinib resistance.

Detection of compound mutations is more frequent among CML-BP and Ph+ ALL patients compared to those with CML-CP, thus suggesting an increased risk of compound mutation-based multidrug resistance in advanced disease.^[10]^ Immunotherapy, such as chimeric antigen receptor T-cells therapy, is considered a treatment option with the potential to overcome TKIs resistance based on ABL mutations.^[11–13]^ Unfortunately, the blinatumomab and inotuzumab ozogamicin are not available for Chinese patients because they have still not been approved by CFDA. Poor physical condition and multiple complications limited our patient from enrolling chimeric antigen receptor T-cells clinical trials. Finally, Y253H/E255K(V) and other possible compound mutations were fatal to the patient. Recently, the allosteric inhibitor asciminib, which was combined with ponatinib, showed exciting ability to suppress emergence of and restore efficacy against highly resistant BCR-ABL1 compound mutants.^[14]^ Thus, asciminib-based drug combinations may offer a novel treatment option to overcome TKIs resistance in Ph+ ALL.

In conclusion, second and third-generation TKIs could be effective in patients with recurrent Ph+ ALL, while multidrug-resistant compound mutations within the BCR-ABL1 kinase domain are an emerging clinical problem for patients receiving sequential TKIs therapy. Therefore, it is of urgent importance to consider HSCT or novel immunotherapies and differentiate compound mutations from polyclonal mutations in order to come up with the best treatment option. Acquisition of E255K/V-inclusive mutations is usually associated with ponatinib resistance, thus it is necessary to screen out new real pan-inhibitor compounds for all BCR/ABL mutations and figure out the potential efficacy of asciminib-based drug combinations in the future.

(Supplementary material.doc).

## Author contributions

**Conceptualization:** Miaomiao Zhao, Qiuling Wu, Linghui Xia.

**Data curation:** Miaomiao Zhao, Xiya Gui.

**Project administration:** Yadan Wang.

**Writing – original draft:** Miaomiao Zhao, Xiya Gui, Qiuling Wu.

**Writing – review & editing:** Miaomiao Zhao, Xiya Gui, Qiuling Wu, Linghui Xia, Yadan Wang.

## Supplementary Material

Supplemental Digital Content

## Supplementary Material

Supplemental Digital Content

## Supplementary Material

Supplemental Digital Content
