# Supplementary material for: The role of E255K/V-inclusive mutations in a Philadelphia-positive acute lymphoblastic leukemia with mutation evolution during sequential TKIs therapies: A case report
Source: Medicine (Baltimore). 2021 May 7;100(18):e25579. doi: 10.1097/MD.0000000000025579 (PMC8104221; doi:10.1097/MD.0000000000025579)

**Additional file 1: Figure S1 The structural changes caused by the resistance of single point mutations (E255K, E255░V, and Y253H) to second- and third-generation TKIs.** TKIs were displayed in wheat sticks. ABL kinase is illustrated in pale green cartoon representation, and the interacting residues are labeled and shown as green sticks (wild type) and cyan sticks (mutat type). Interactions between residues and drugs are donated in dashed lines: hydrogen bondings in blue, salt bridge in red, π-π stacking in yellow, and hydrophobic interaction in gray.


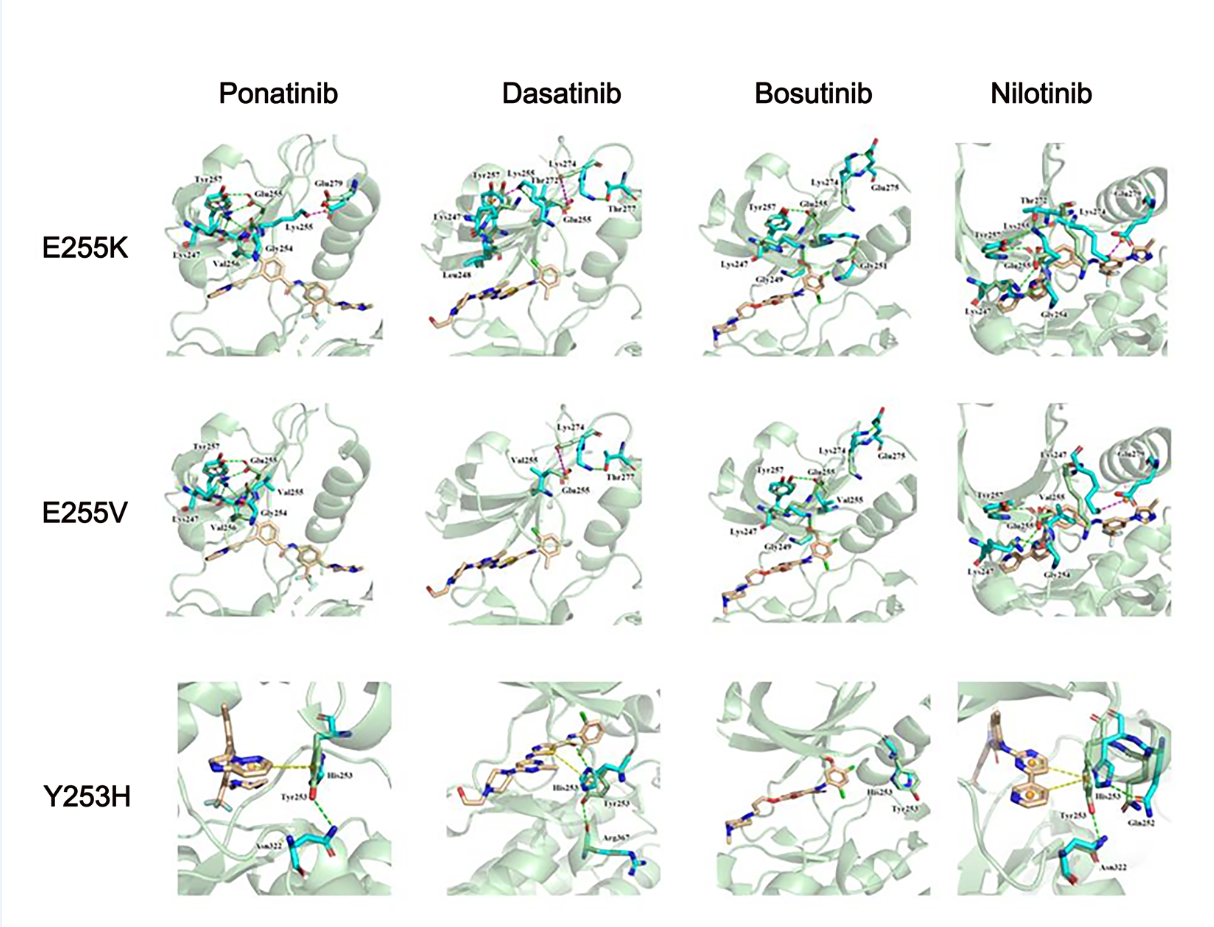

Supplement: Supplemental Digital Content [file medi-100-e25579-s001.doc]
