# Supplementary material for: The role of E255K/V-inclusive mutations in a Philadelphia-positive acute lymphoblastic leukemia with mutation evolution during sequential TKIs therapies: A case report
Source: Medicine (Baltimore). 2021 May 7;100(18):e25579. doi: 10.1097/MD.0000000000025579 (PMC8104221; doi:10.1097/MD.0000000000025579)
Supplement: Supplemental Digital Content [file medi-100-e25579-s002.doc]

**Table S1. The resistance indices of single and compound mutations to four TKIs**

| mutations | Bosutinib | Dasatinib | Nilotinib | Ponatinib | source |
| --- | --- | --- | --- | --- | --- |
| PDB code | 3UE4 | 2GQG | 3CS9 | 3IK3 | RCSB Protein Data Bank |
| Resolution (Å) | 2.424 | 2.4 | 2.21 | 1.9 |  |
| Y253H | 0 | 0 | 3 | 0 | Ref 9, 10 |
| E255V | 1 | 0 | 3 | 2 |  |
| E255K | 2 | 2 | 2 | 2 |  |
| Y253H/E255V | **1** | 0 | 6 | **2** |  |
| Y253H/E255K | 2 | 2 | 5 | 2 |  |

A color gradient from green (sensitive) to yellow (moderately resistant) to orange (resistant) to red (highly resistant) denotes the sensitivity to each TKI, which has been reported in previous studies.

RI, resistance index

RI = 0: the mutation did not affect drug binding and was reported to be sensitive;

RI = 1: the mutation slightly weakened protein-drug interactions, which had some effect on drug binding, corresponding to moderate resistance in the literature;

RI = 2: the mutation reduced protein-drug interactions, which had a significant impact on drug binding, corresponding to resistance in the literature;

RI = 3: the mutation caused conformational changes or deficiency of protein-drug interactions, which significantly influenced drug binding, corresponding to high resistance in the literature.
