# Supplementary material for: The role of E255K/V-inclusive mutations in a Philadelphia-positive acute lymphoblastic leukemia with mutation evolution during sequential TKIs therapies: A case report
Source: Medicine (Baltimore). 2021 May 7;100(18):e25579. doi: 10.1097/MD.0000000000025579 (PMC8104221; doi:10.1097/MD.0000000000025579)
Supplement: Supplemental Digital Content [file medi-100-e25579-s003.doc]

**Supplementary information**

**Analysis of Single Point Mutation**

The crystal structures of ABL kinase and drug complexes were downloaded from the RCSB Protein Data Bank (Bosutinib: 3UE4, Dasatinib: 2GQG, Nilotinib: 3CS9, and Ponatinib: 3IK3). Based on the drug activity data reported in the literature ^1,2^ and structural comparison of the native and mutated proteins, the mechanism of resistance caused by single point mutations was analyzed (**Figure S1**).

**Ponatinib**

Y253H: The benzene ring of Tyr253 formed a π-π stacking interaction with the imidazolidine ring of Ponatinib, while the phenolic hydroxyl group formed a hydrogen bond with Asn322. With the residue mutated to His253, the aromatic ring became smaller. Although the hydrogen bonding was lost, π-π stacking remained, effectively preventing drug resistance (resistance index (RI) = 0).

E255V: In the native protein, Glu255 interacted with Tyr257 and Lys247 with two hydrogen bonds, contributing to a specific conformation of the P-loop. In E255V mutation, the hydrogen bond with Val255 was lost, but Lys247 formed hydrogen bonds with the backbone oxygen atoms of both Gly254 and Val256, and also formed a π-cation interaction with the benzene ring of Tyr257, which changed the original conformation of the P-loop, resulting in a certain degree of drug resistance (RI = 2).

E255K: Similar to E255V, in this mutation, Lys247 formed the same interactions with surrounding residues Gly254, Val256, and Tyr257; but Lys255 with a long side chain and protonated N atom was able to interact with Glu279 by a salt bridge, which also changed the original conformation of P-loop (aa. 244-255) ^3^, resulting in drug resistance (RI = 2).

**Bosutinib**

Y253H: No direct contact was observed between Tyr253 and Bosutinib. Hence, when it was mutated to His253, the impact on Bosutinib was relatively small. Therefore, no drug resistance was generated for ABL kinase (RI = 0).

E255V: In the native protein, Glu255 formed hydrogen bonds with both Tyr257 and Lys247 in the P-loop region, which play an essential role in maintaining the conformation of P-loop. The mutation of E255V brought about a loss of these two interactions and the orientation of Lys247 towards Gly249, forming a hydrogen bond. Moreover, Lys274 underwent a significant conformational change, producing a hydrogen bond with Glu275. These might induce a conformational change of the P-loop and affect the binding of Bosutinib. Resistance was reported in reference 1, while sensitivity in reference 2. According to the above analyses, we concluded that it would lead to moderate drug resistance (RI = 1).

E255K: This mutation was similar to E255V. In addition to the loss and gain of the hydrogen bonds mentioned above, Lys255 can form hydrogen bonds with Gly251 located in the turning of the P-loop. Such interaction changes might cause considerable conformational alteration in P-loop, leading to drug resistance (RI = 2).

**Dasatinib**

Y253H: In the ABL kinase-Dasatinib complex, Dasatinib through the S atom directly interacted with the benzene ring of Tyr253 by a π-S interaction ^4^ Meanwhile, a hydrogen bonding took place between the hydroxyl group of Tyr253 and Arg36, which had a significant effect on stabilizing the conformation of P-loop. It made a certain contribution to the binding of the drug Dasatinib. In Y253H mutation, His253 lost the hydrogen bond to Arg367 due to its smaller volume of the ring moiety and loss of the phenolic hydroxyl group as a hydrogen-bonding donor, which might magnify conformational instability of the P-loop region. However, a strong hydrogen bond of the amide O atom in Dasatinib to His253 kept it from the resistance of the protein (RI = 0).

Notably, previous molecular dynamics (MD) simulation results ^2^ suggested that the amide bond of Dasatinib flipped over to form a hydrogen bond with His253. However, in this study, we found that the amide bond of Dasatinib maintained the original conformation and interaction, i.e., the hydrogen bond of the amide N atom of Dasatinib to Thr315 (not shown in **Figure S1**). After flipping, the amide O atom face the backbone O atom of Glu316 (not shown in **Figure S1**). In this case, electrostatic repulsion occurs. Therefore, this study adopted a different hydrogen-bonding mode compared to that shown in the literature.

E255V: The carboxyl group of Glu255 being an anion center formed a salt bridge with the N atom, the cation center, of Lys274. The E255V mutation deprived this interaction. Meanwhile, Lys274 deflected towards Thr277, forming a hydrogen bond between one another. Yet, Val255 is a small-volume neutral residue, thus was not totally affected by the changes in the interaction. As a result, the conformation of the P-loop and binding interactions of the drug molecule were not affected. Furthermore, the mutant protein was still sensitive to Dasatinib (RI = 0).

E255K: Unlike E255V, Lys255 was a long-chain residue with positive charges, which could quickly turn to Tyr257 and Thr272, forming π-cation interaction and hydrogen bonding, respectively. Under the electrostatic repulsion of Lys255, Lys247 and Lys274 turned to form hydrogen bonds with Leu248 and Thr277, respectively. Leu248 and Lys255 were both located in the P-loop region. In such a complex interaction network, the P-loop was induced to undergo a conformational change, which significantly affected the drug binding and the protein exhibited drug resistance (RI = 2).

**Nilotinib**

Y253H: Tyr253 showed a π-π stacking interaction with the 4-(pyridin-3-yl) pyrimidine moiety of Nilotinib, and also formed a hydrogen bond with Asn322 that was important in maintaining the P-loop conformation. In Y253H mutation, the original hydrogen bond was lost while a new one between His253 and Gln252 was obtained, by which this mutated residue was induced away from the 4-(pyridin-3-yl) pyrimidine moiety of Nilotinib and lost the π-π stacking interactions. As a result, the protein-drug interactions were diminished, leading to drug resistance (RI = 3).

E255V: Glu255 formed hydrogen bonds with Lys247 and Tyr257 simultaneously through the carboxyl group, which was crucial in maintaining the P-loop conformation. In E255V mutation, these two interactions were both lost, leading to a hydrogen bond of Lys247 to Gly254 and a salt bridge with Glu279. This mutation affected the conformation of the P-loop, and in turn, affected the binding affinity of Nilotinib (RI = 3).

E255K: Unlike E255V, the mutant Lys255 resided among Tyr257, Thr272, and Lys274, forming hydrogen bonds, which mitigated the conformational change of P-loop caused by the mutation. Therefore, the protein produced specific drug resistance, and yet less than the impact of E255V (RI = 2).

**Prediction of Compound Mutation**

Based on the drug resistance index of single point mutation, we calculated the drug resistance index of compound mutation and predicted its impact (**Table S1**), among which some compound mutations have been reported in the literature. ^1,2^

**Y253H/E255V**

The single point mutation of E255V is sensitive to Dasatinib (RI = 0), moderately resistant to Bosutinib (RI = 1), strongly resistant to Ponatinib (RI = 2), and highly resistant to Nilotinib (RI = 3). Therefore, the compound mutation Y253H/E255V is only sensitive to Dasatinib (RI = 0), moderately resistant to Bosutinib (RI = 1), and highly resistant against both Nilotinib (RI = 6) and Ponatinib (RI = 2). As residues 253 and 255 were both located in the P-loop, once mutated, a synergistic effect could be seen. It severely altered the conformation of P-loop and affected the binding site of the drug, which thereby generated drug resistance beyond any single point mutation. As illustrated in reference, ^2^ molecular dynamics simulation studies showed that the compound mutation triggered a remarkable shift of P-loop, affected the binding site of Ponatinib, and lost the π-π stacking between Tyr253 and Phe382. Phe382 was also pushed into the binding site of Ponatinib and blocked the key salt bridge of Lys271 with Glu286. This confirmed the capacity of the drug resistance index in the evaluation of drug resistance of compound mutations.

**Y253H/E255K**

As E255K showed resistance against all drugs, although Y253H was sensitive to Bosutinib, Dasatinib, and Ponatinib, the compound mutation still had resistance. In particular, Y253H was highly resistant against Nilotinib, and the compound mutation further reinforced it. Therefore, the Y253H/E255K mutation demonstrated moderate or high resistance against all the four drugs.

**References**

1. Redaelli S, Mologni L, Rostagno R, et al. Three novel patient-derived BCR/ABL mutants show different sensitivity to second and third generation tyrosine kinase inhibitors. *American journal of hematology.* 2012;87(11):E125-128.

2. Zabriskie MS, Eide CA, Tantravahi SK, et al. BCR-ABL1 compound mutations combining key kinase domain positions confer clinical resistance to ponatinib in Ph chromosome-positive leukemia. *Cancer cell.* 2014;26(3):428-442.

3. Jabbour E, Kantarjian H, Jones D, et al. Frequency and clinical significance of BCR-ABL mutations in patients with chronic myeloid leukemia treated with imatinib mesylate. *Leukemia.* 2006;20(10):1767-1773.

4. Tanneeru K, Guruprasad L. Ponatinib is a pan-BCR-ABL kinase inhibitor: MD simulations and SIE study. *PloS one.* 2013;8(11):e78556.
